# Tumor resection in paramedian structures of the frontal lobe poses a risk for corpus callosum infarction

**DOI:** 10.1007/s00701-025-06555-y

**Published:** 2025-05-13

**Authors:** Yoshiteru Shimoda, Masayuki Kanamori, Shinichiro Osawa, Shingo Kayano, Ryuta Saito, Mugikura Shunji, Tominaga Teiji, Hidenori Endo

**Affiliations:** 1 Department of Neurosurgery, Tohoku University Graduate School of Medicine, 1-1 Seiryo-machi Aoba-ku, Sendai, 980-8574 Japan; 2 Department of Radiological Technology, Tohoku University, Tohoku University, HospitalSendai, Miyagi Japan; 3 Department of Neurosurgery, Nagoya University Graduate School of Medicine, Nagoya, Japan; 4 Department of Diagnostic Radiology, Tohoku University Graduate School of Medicine, Sendai, Japan; 5 Department of Image Statistics, Tohoku Medical Megabank Organization, Tohoku University, Sendai, Japan

**Keywords:** Cingulate gyrus, Surgery, Disconnection syndrome, Corpus callosum

## Abstract

**Purpose:**

Surgeons resecting intraparenchymal tumors should be aware of potential white matter ischemia resulting from damage to the medullary artery arising from the cerebral cortex. In the vicinity of the paramedian structure, crucial brain regions for higher brain function such as corpus callosum and cingulate cortex are located. However, the actual area of ischemia induced by damaging the medullary artery supplying the paramedian structures is not known. The present study investigated the ischemic field following tumor resection in paramedian structures of the frontal lobe.

**methods:**

Patients having intraparenchymal tumors with lesions in the paramedian structures of the frontal lobe (superior frontal gyrus or cingulate gyrus) resected between April 2016 and June 2022 at Tohoku University Hospital were included in the study. Magnetic resonance images obtained within 72 h after surgery were used to retrospectively examine the extent of the resection and the distribution of ischemic complications. Related postoperative clinical symptoms were assessed using medical records.

**Results:**

Thirty-three cases matched the inclusion criteria. The median age was 48 years. Cases comprised patients with an astrocytoma IDH-mutant (*n* = 11), oligodendroglioma IDH-mutant, and 1p/19q-codeletion (*n* = 12), and glioblastoma IDH-wildtype (*n* = 10). The main locations were superior frontal gyrus only (*n* = 17), cingulate gyrus only (*n* = 8), and both the frontal lobe and cingulate gyrus (*n* = 8). The cingulate gyrus was removed in 19 cases. In 16 of the 19 cases, ischemic foci were observed in the adjacent corpus callosum. In the 14 cases in which the cingulate gyrus was not removed, no ischemic foci appeared in the corpus callosum. Three cases exhibited a prolonged disturbance of consciousness after the second postoperative day, all with corpus callosum infarction.

**Conclusion:**

Surgeons resecting intraparenchymal tumors in the paramedian structures of the frontal lobe, especially the cingulate gyrus, should be aware of the potential for ischemia foci emerging in the corpus callosum.

## Introduction

The corpus callosum is a crucial structure connecting the left and right hemispheres of the brain. The callosal artery, which branches directly from the pericallosal artery, feeds the midline region of the corpus callosum. The corpus callosum extends laterally into the deep white matter, where branching vessels from the callosal sulcus supply the transition zone between the corpus callosum and the deep white matter [14]. Branches from the pericallosal artery travel through the callosal sulcus and supply feeding vessels to the corpus callosum, including the callosal, cingulocallosal, short callosal, long callosal, and direct perforating callosal arteries [3, 5, 6, 11, 14].

Detailed microangiographic studies by Okudera[9] and Akashi[1] revealed that arteries perforating the cingulate gyrus partially supply the lateral part of the corpus callosum where it transitions into the deep white matter. These findings, in contrast to previous assumptions, indicate that the corpus callosum receives its blood supply not only from the callosal sulcus but also from the medullary artery arising from the cortex of the paramedian structures.

Based on these observations, we hypothesized that resection of the cingulate gyrus could pose a risk for ischemic injury to the cerebral corpus callosum. In this study, we applied imaging techniques and clinical evaluation to investigate the risk of injury to the corpus callosum when the cingulate gyrus is resected during intraparenchymal tumor surgery.

## Methods

This retrospective study was approved by the Institutional Review Board of Tohoku University Hospital (2023–1–416). Due to the retrospective nature of the study, patient consent was waived, though patients were provided the opportunity to opt out of the study.

We included intraparenchymal tumors with lesions in the superior frontal gyrus or cingulate gyrus (anterior cingulate cortex, midcingulate cortex, and posterior cingulate cortex[15]) that were resected between April 2016 and June 2022 at Tohoku University Hospital. Tumor locations were confirmed by preoperative magnetic resonance imaging (MRI), and maximal safe resection was performed.

Postoperative MRI scans were performed within 72 h after surgery to assess ischemic lesions. The extent of the resection was confirmed using T1-weighted 3D images. The distribution of ischemia was assessed with diffusion-weighted imaging. Postoperative clinical symptoms were retrospectively collected from the medical records.

To determine the anatomic relationship between the extent of resection and the extent of infarction, the long-axis length of the resected cingulate gyrus and the length of the ischemic area in the corpus callosum were measured in postoperative magnetic resonance images. The distance from the posterior border of the genu of the corpus callosum was calculated from T2-weighted and diffusion-weighted sagittal images. To analyze the relationship between the extent of ischemia in the corpus callosum and the extent of the cingulate gyrus resection, we calculated the ratio of overlap in the long axis to the extent of the ischemic area in the corpus callosum.

### Surgical procedure

During the resection of the cingulate gyrus, great care was taken to avoid injury to the arteries running through the callosal sulcus. A subpial resection was primarily performed. However, when the pia mater was weak and prone to injury, extra attention was paid to avoid damaging the small vessels attaching to the corpus callosum while gently lifting the cingulate gyrus.

## Results

Thirty-three cases matched the inclusion criteria. Median patient age was 48 (range, 24–75) years. Of the 33 cases, there were 11 cases of astrocytoma IDH-mutant, 12 cases of oligodendroglioma IDH-mutant with 1p/19q-codeletion, and 10 cases of glioblastoma IDH-wildtype. The tumor was confined to the frontal lobe in 17 cases, the cingulate gyrus in 8 cases, and both the frontal lobe and cingulate gyrus in 8 cases.

**Fig. 1:**
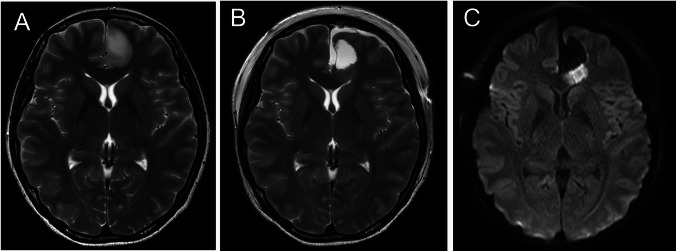
Typical ischemic lesion after cingulate gyrus resection without injury to the corpus callosum. A 24-year-old woman underwent MRI for evaluation of headaches. An enlarged T2 high-signal area was observed in the anterior cingulate gyrus (**A**). A low-grade glioma was suspected, and total resection was performed without injury to the corpus callosum (**B**). Postoperative MRI showed an ischemic area in the corpus callosum adjacent to the resected area (**C**). Postoperatively, the patient exhibited no new neurologic symptoms

**Fig. 2:**
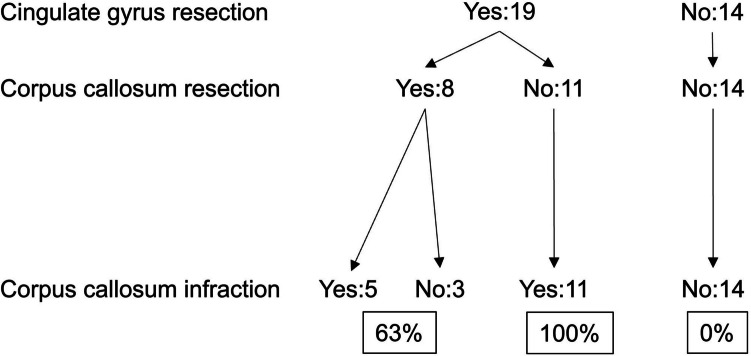
Cingulate gyrus resection results in corpus callosum infarction. The cingulate gyrus was removed in 19 cases. In 8 of these cases, the corpus callosum was also partially resected. In these 8 cases, 5 exhibited ischemia of the corpus callosum (63%). In the 3 cases with corpus callosum ischemia, the corpus callosum directly below the resected cingulate gyrus was also removed, making it difficult to evaluate ischemic lesions of the corpus callosum. In 11 cases, the cingulate gyrus was removed but the corpus callosum was completely preserved. In all 11 cases, ischemia was observed at the lateral side of the corpus callosum (100%). In contrast, in 14 cases in which the cingulate gyrus was not resected, no ischemia of the corpus callosum was observed (0%)

The cingulate gyrus was removed in 19 cases. Clinical information for the cingulate gyrus resection and non-resection groups is summarized in the Table 1. In 16 (84.2%) of these 19 cases, ischemia was observed in the adjacent corpus callosum A representative case is shown in Fig. 1. In the remaining 3 cases, the corpus callosum directly below the resected cingulate gyrus was also removed, making it difficult to evaluate ischemic lesions in the corpus callosum. In 11 cases, the cingulate gyrus was resected but the corpus callosum was completely preserved. In all of these cases, ischemia was observed on the lateral side of the corpus callosum (100%). In contrast, among the 14 cases in which the cingulate gyrus was not resected, no ischemia was observed in the corpus callosum (0%). These results are summarized in Fig. 2.

**Table 1:** Patient background and outcome after cingulate gyrus resection

	All	Cingulate gyrus resection	Cingulate gyrus non-resection	p
	33	19 (57.6)	14 (42.4)	
Sex Male (%)	18 (54.6)	11 (57.9)	7 (50)	0.7
Age, median (range)	48 (24–75)	45 (24–75)	50 (29–73)	
Histology				
Glioblastoma (%)	10 (29.4)	6 (31.6)	4 (28.6)	
Diffuse astrocytoma (%)	4 (11.8)	3 (15.8)	1 (7.1)	
Anaplastic astrocytoma (%)	7 (20.6)	3 (15.8)	4 (28.6)	
Oligodendroglioma (%)	9 (26.5)	5 (26.3)	4 (28.6)	
Anaplastic oligodendroglioma (%)	3 (8.8)	2 (10.5)	1 (7.1)	
Tumor location				
Side (Lt) (%)	11 (33)	8 (42)	3 (21)	0.28
Main location				
Frontal lobe	17	14 (73.7)	3 (21.4)	
Cingulate gyrus	8	8 (42.1)	0 (0)	
Frontal lobe and cingulate gyrus	8	8 (42.1)	0 (0)	
Including cingulate gyrus	19	16 (84.2)	3 (21.4)	< 0.0001
After surgery				
Corpus callosum infarction	16 (47)	16 (84.2)	0 (0)	< 0.0001
Delay of consciousness disturbance				
More than 1 days	8 (24.2)	7 (36.8)	1 (7.1)	0.098
More than 3 days	3 (9.1)	3 (15.8)	0 (0)	0.24

**Fig. 3:**
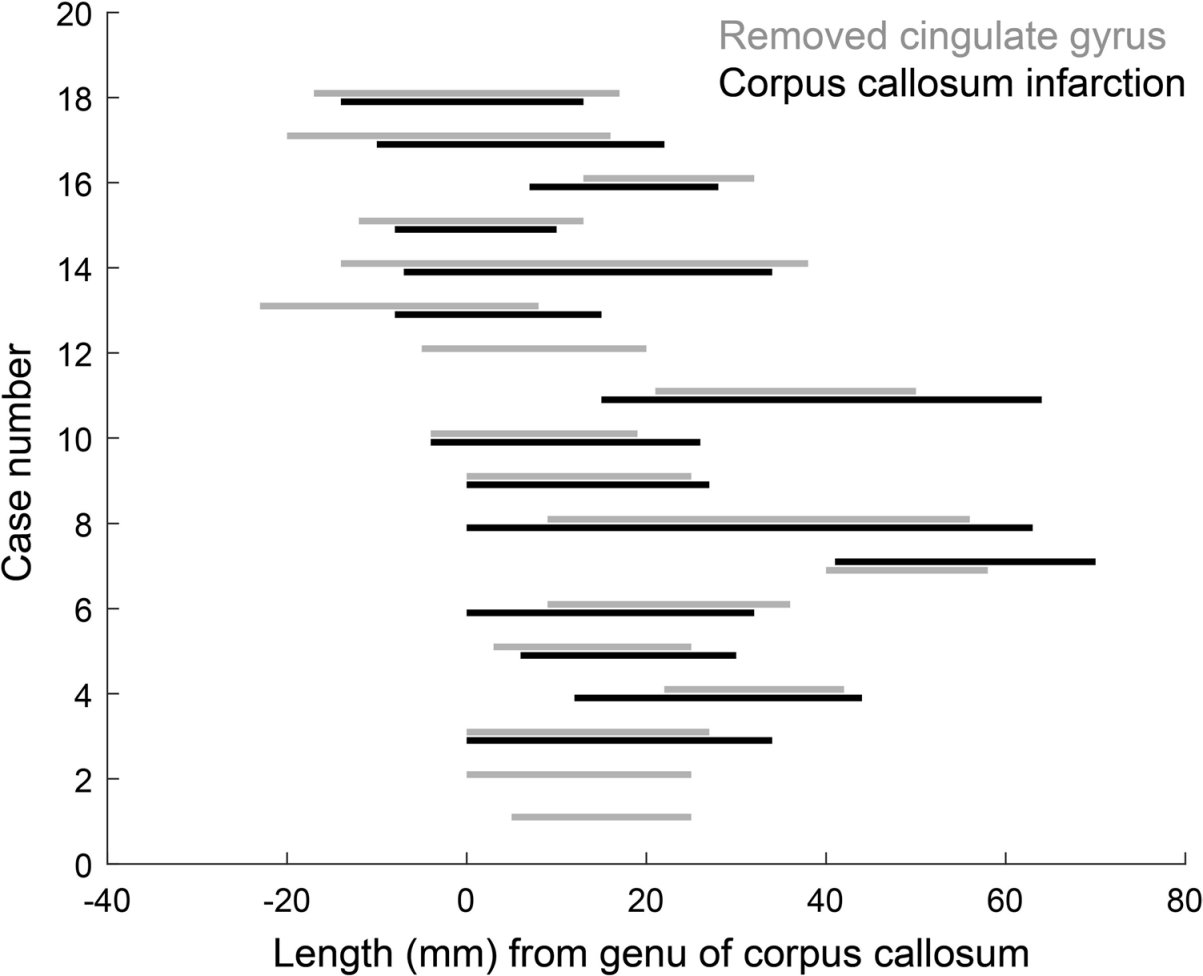
Extent of the ischemic region in the corpus callosum plotted coaxially with the extent of the cingulate gyrus resection

If part of the corpus callosum is supplied by terminal vessels via the cingulate gyrus, then the extent of the ischemic region should coincide with the extent of the cingulate gyrus resection. The extent of the ischemic region in the corpus callosum was plotted coaxially with the extent of the cingulate gyrus resection (Fig. 3). The mean length of the cingulate resection was 26.3 (1.2) mm [mean (SD)]. The mean length of the ischemic region or resection of the corpus callosum was 31 (3.6) mm. The results indicated that 81% of the ischemic region in the anterior–posterior extent of the corpus callosum coincided with the resected area of the cingulate gyrus. Furthermore, we examined the relationship between the location of cingulate gyrus resection and the occurrence of ischemic lesions in the corpus callosum. Given that the length of the corpus callosum is approximately 10 cm, we classified the regions into three parts: Anterior (−1 to 2 cm of Fig. 3), Middle (2 to 5 cm of Fig. 3), and Posterior (5 to 8 cm of Fig. 3). We then calculated the ratio of the ischemic occurrence distance to the resection distance in these three regions. The frequency of ischemic lesions in the corpus callosum corresponding to each resection area was 97% for the Anterior, 114% for the Middle, and 135% for the Posterior regions. These results suggest that ischemia tends to extend beyond the resection site, particularly posteriorly. This may be influenced by the fact that resections were primarily performed anteriorly, and fewer cases involved resection of the posterior cingulate gyrus.

**Fig. 4:**
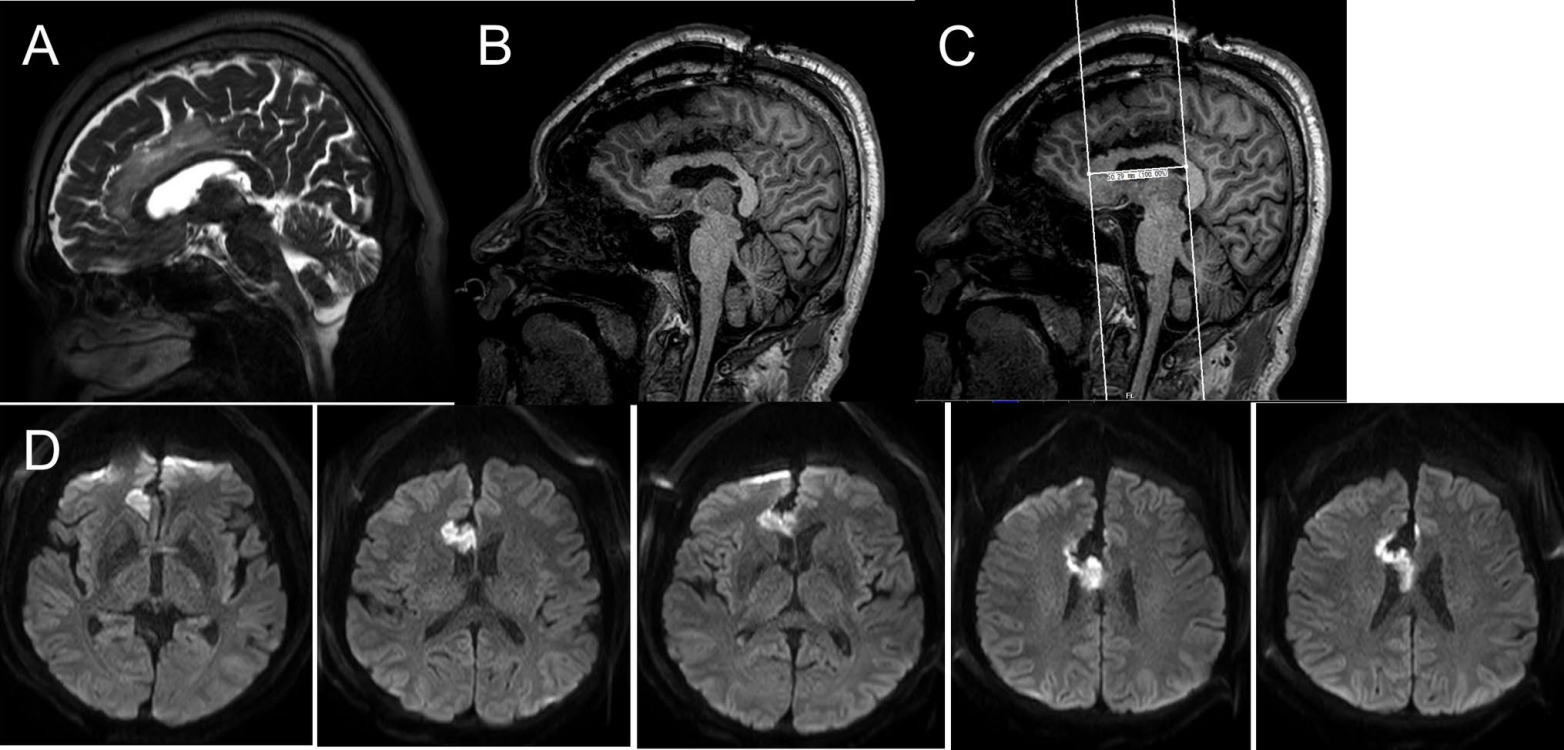
A 67-year-old man underwent an incidental MRI. The cingulate gyrus was enlarged, extended along the long axis, and exhibited a high T2 signal (**A**). A low-grade glioma was suspected and resection was performed. The entire high T2 signal area was removed (**B**). The resection extended 5 cm longitudinally (**C**). Postoperative MRI showed an ischemic area in the corpus callosum lateral to the resected region (**D**). Postoperatively, he exhibited consciousness disturbances for 7 days

**Fig. 5:**
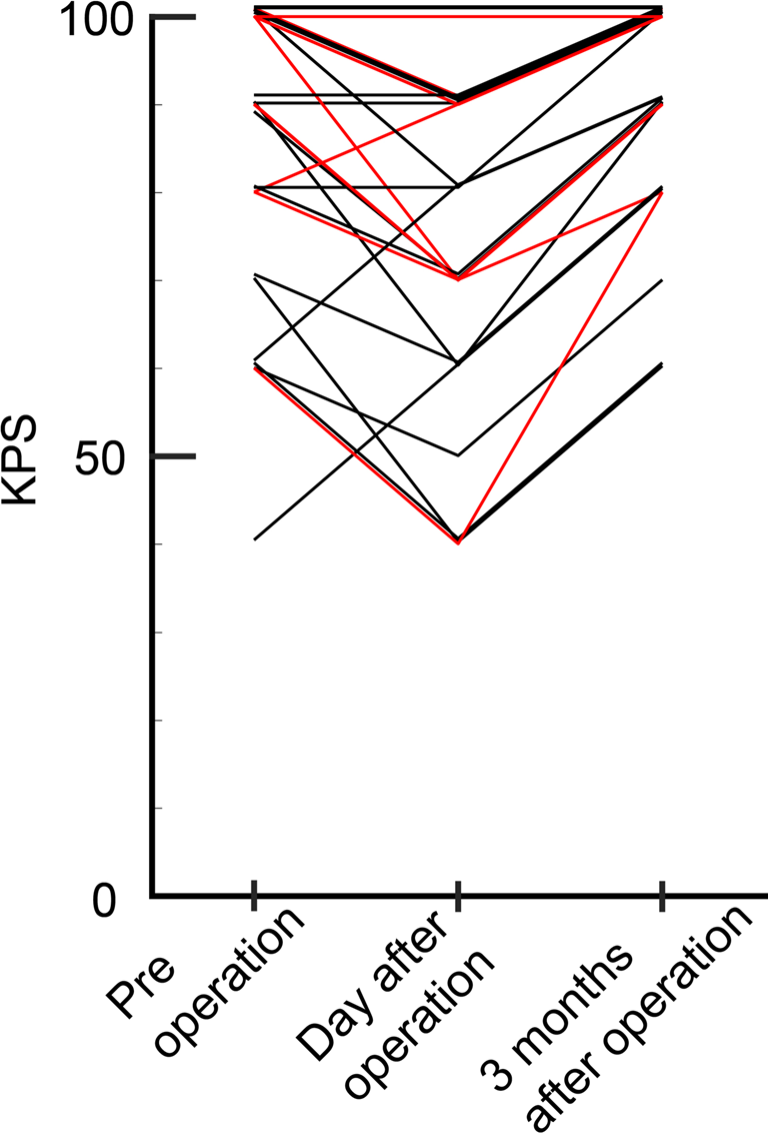
The temporal changes in Karnofsky performance scale (KPS) in the group of cases with no lesions in the cingulate gyrus that caused no corpus callosum infarction (black line) and the group of cases with lesions in the cingulate gyrus that caused corpus callosum infarction due to cingulate gyrus removal (red line), before and at day after surgery, and at 3 months after surgery. The data for the 2 patients who had had a relapse at 3 months, were excluded. In both groups, KPS temporarily decreased after surgery, but tended to recover by 3 months after surgery. There was no difference in the degree of recovery between the two groups (*p* = 0.19)

In 8 cases, consciousness disturbances lasting more than 1 day were noted after surgery. Postoperative impaired consciousness tended to be more common in cases in which the cingulate gyrus was removed (36.8% for the cingulate gyrus resection group vs 7.1% for the cingulate gyrus non-resection group, *P* = 0.098) Among these 8 cases, 3 had prolonged disturbed consciousness for more than 3 days, and all 3 cases had ischemic lesions in the corpus callosum (15.8% in the cingulate gyrus resection group vs 0% for cingulate gyrus non-resection group, *P* = 0.24). One case (Case 2, Fig. 4) in which the corpus callosum was not touched had a long ischemic lesion in the corpus callosum immediately below the resected cingulate gyrus. Some cases with impaired consciousness had ischemic foci extending as far back as the splenium of the corpus callosum. Disturbed consciousness continued in 2 cases until postoperative day 4 and in 1 case until postoperative day 6. The Karnofsky performance scale (KPS) were compared between the group of patients who had not had the cingulate gyrus removed and the group of patients who had had ischemia at the corpus callosum after having had the cingulate gyrus removed, both before and at day after surgery and at 3 months after surgery (Fig. 5). The data for 2 patients who had had a relapse before 3 months were excluded. In both groups, KPS temporarily decreased after surgery, but tended to recover by 3 months after surgery. There was no difference in the degree of recovery between the two groups (*p* = 0.19).

## Discussion

In all cases of cingulate gyrus resection without damage to the corpus callosum, ischemia was observed in the corpus callosum. In some cases, cingulate gyrus resection-related ischemia of the corpus callosum resulted in symptomatic complications.

### Mechanism of ischemia in the corpus callosum following cingulate gyrus resection

Ischemia of the corpus callosum was observed after removing the cingulate gyrus, while ischemia of the corpus callosum was not observed in cases in which the cingulate gyrus was not resected. To our knowledge, no previous studies have pointed out the relationship between cingulate gyrus resection and ischemia of the corpus callosum.

**Fig. 6:**
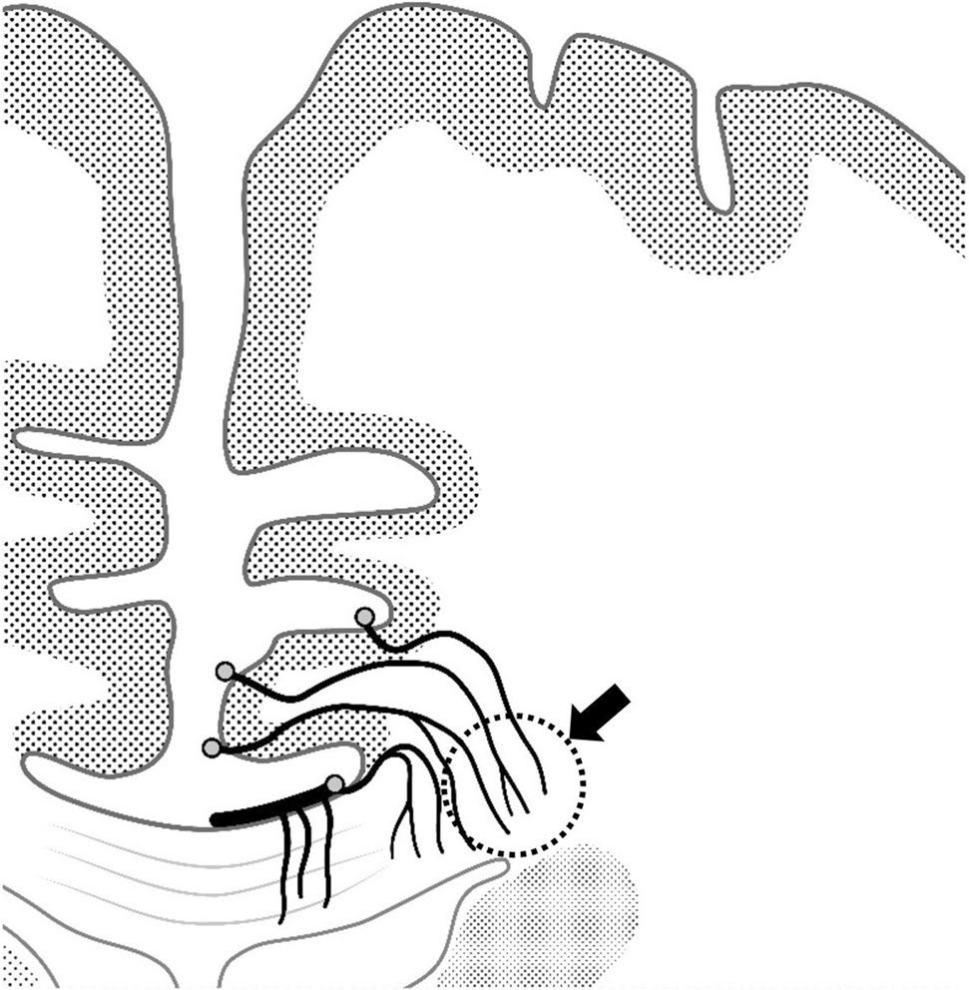
Microvascular images of the corpus callosum obtained from the literature. Modified from T Okudera et al. Neuropathology 1999[9]. Note that the perforating artery supplying the outer part of the corpus callosum runs through the cingulate gyrus (arrow), suggesting that infarction of the corpus callosum is inevitable when the cingulate gyrus is resected

**Fig. 7:**
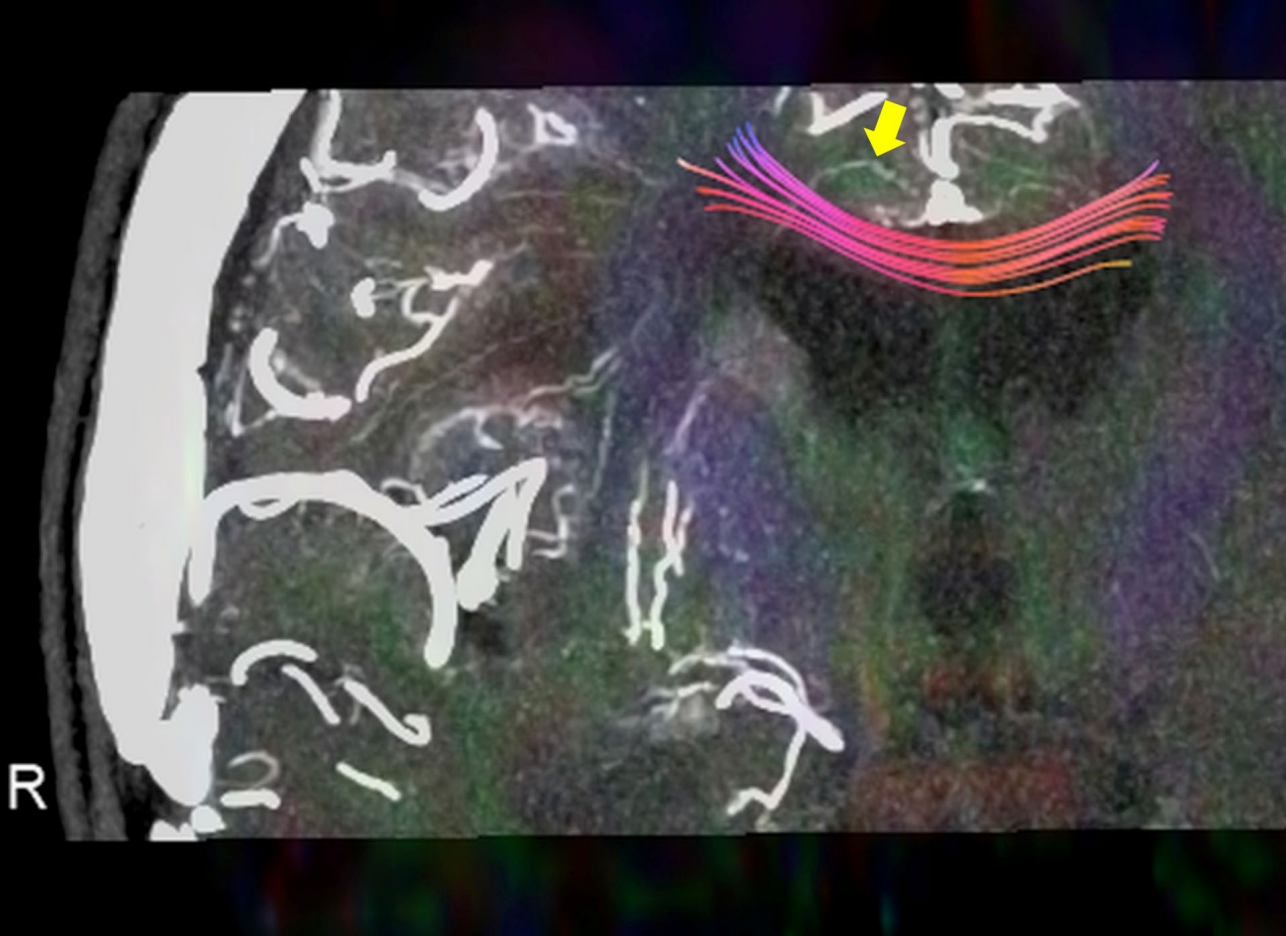
The combination of intra-arterial administration of contrast medium and ultrahigh-resolution computed tomography angiography allowed for observation of the perforating artery supplying the outer part of the corpus callosum running through the cingulate gyrus (arrow). The 3D tractography of the corpus callosum, derived from diffusion tensor imaging, is being fused

Previous microangiographic studies demonstrated that the medullary artery, which runs within the cingulate gyrus, partially supplies the corpus callosum[9] (Fig. 6). Similar results were observed in other cases evaluated by a method combining intraarterial administration of contrast medium and ultrahigh-resolution computed tomography angiography[10] (Fig. 7). Our findings confirmed this anatomic observation, as ischemia appeared in all 11 cases in which the cingulate gyrus was resected without directly manipulating the corpus callosum, indicating minimal anatomic variation in this respect. Further, we noted that the infarcted area tended to expand not only lateral to the resected area, as previously reported[14], but also in the anteroposterior direction. Although we were unable to find previous studies specifically examining the vascular architecture in the anteroposterior direction of the corpus callosum, it is possible that the medullary artery provides a large anteroposterior blood supply to the corpus callosum after it passes through the cingulate gyrus. Understanding this anatomic mechanism is crucial for clinicians performing surgical procedures and considering the extent of tissue removal.

### Risk of corpus callosum ischemia following cingulate gyrus removal

While cingulate gyrus resection is generally considered safe, the present study revealed an associated risk of inducing ischemia in the corpus callosum. Tate et al.[12] reported on the risks associated with cingulate gyrus resection[12], describing 34 cases in which only the cingulate gyrus was resected. In 9 of these cases, neurologic deficits appeared postoperatively, mostly due to supplementary motor area (SMA) syndrome, with damage observed in the SMA proper along the surgical trajectory. They did not, however, mention symptoms of corpus callosum disconnection. Their evaluation relied solely on standard clinical examinations, and they acknowledged that the possibility of disconnection symptoms could not be ruled out.

The corpus callosum connects the left and right hemispheres of the brain. Damage to this structure can result in acute disconnection syndrome [4], characterized by temporary disturbance of consciousness, bilateral Babinski responses, forced grasping with the nondominant hand, nondominant hand apraxia to verbal commands, and symptoms similar to SMA syndrome, including incontinence, paresis of the nondominant arm and leg, and speech difficulties [4, 8, 16]. In most cases, however, these symptoms are absent altogether or improve rapidly [16].

Two clinical situations are particularly important. First, when the cingulate gyrus is resected over a long segment, as in Case 2 (Fig. 4), consciousness impairment is more likely as the corpus callosum is also extensively damaged [8, 16]. Prolonged postoperative consciousness disruption can greatly impact treatment planning and timing, especially for elderly patients or those with malignancies. If the cingulate gyrus is resected longitudinally, the adjacent area of the corpus callosum may become ischemic. Therefore, it is important to recognize that longitudinal resection of the cingulate gyrus may lead to postoperative consciousness disturbances.

Second, when the posterior cingulate gyrus is resected, ischemia can occur in the splenium of the corpus callosum, resulting in long-lasting visuo-hemispatial neglect, left visual field alexia, and anomia.[2, 7, 13]

Based on the present review and previous literature, corpus callosum infarction appears to be an unavoidable complication when the cingulate gyrus is removed. Consequently, clinicians must be aware of the clinical risks associated with cingulate gyrus resection, particularly in these situations.

### Limitations

The present study was a retrospective study. Methods to assess the vascularization of the corpus callosum preoperatively are not currently available. Such a method could substantiate our findings.

Few reports in the literature have examined variations in the vascular supply of the corpus callosum [9]. Therefore, literature supporting the proposed mechanism is limited, and the possibility of other variations cannot be ruled out.

Despite due precautions, the effects of edema due to venous injury after cingulate resection cannot be ruled out.

Determining whether the impaired consciousness observed in several cases was due to dissection of the cerebral corpus callosum was challenging because resection of the cingulate gyrus always resulted in either the removal of or ischemic damage to the corpus callosum. Therefore, it was impossible to distinguish symptoms caused by damage to the corpus callosum from those caused by resection of the cingulate gyrus.

## Conclusion

Resection of the cingulate gyrus may lead to ischemic lesions in the adjacent corpus callosum. In most cases, these lesions are asymptomatic or recover rapidly. Postoperative sequelae may occur, however, when the ischemic region extends over a long distance or when the posterior part of the cingulate gyrus is resected. Clinicians should be aware of the potential for ischemic foci in the corpus callosum following resection of the cingulate gyrus.

## Data Availability

No datasets were generated or analysed during the current study.
